# The Effect of Fatigue on Trunk and Pelvic Jump-Landing Biomechanics in View of Lower Extremity Loading: A Systematic Review

**DOI:** 10.5114/jhk/159460

**Published:** 2023-01-20

**Authors:** Stefan Vermeulen, Camilla De Bleecker, Cedric De Blaiser, Özge Onursal Kilinç, Tine Willems, Jos Vanrenterghem, Philip Roosen, Roel De Ridder

**Affiliations:** 1 Department of Rehabilitation Sciences, Ghent University, Ghent, East Flanders, Belgium.; 2 Department of Rehabilitation Sciences, KU Leuven, Leuven, Flemish Brabant, Belgium.; 3 Faculty of Physical Therapy and Rehabilitation, Hacettepe University, Ankara, Ankara, Turkey.

**Keywords:** fatigue, trunk, pelvis, landing, biomechanics

## Abstract

Fatigue has often been considered a risk factor for developing sports injuries, modulating lower extremity jump-landing biomechanics. The impact of fatigue on proximal trunk and pelvic biomechanics has been suggested to play an important role in lower extremity loading and injury risk, yet the available evidence remains ambiguous as the trunk and pelvis were often not the primary focus of research. Therefore, the purpose of this systematic review was to determine how fatigue affects trunk and pelvic three-dimensional jump-landing biomechanics. PubMed (MEDLINE), Web of Science, Embase, CINAHL and SPORTDiscus were consulted up to and including April 2022 for potential studies investigating the effect of fatigue on trunk and pelvic kinematics, kinetics and/or muscular activity during jump-landing tasks in healthy, physically active populations. Methodological quality of the studies was assessed by the modified Downs and Black checklist. Twenty-one studies were included and methodological quality was moderate to high among these studies. The results indicate prevailing evidence for more trunk flexion during standardized jump-landing tasks after lower extremity muscle fatigue. Otherwise, lumbo-pelvic-hip muscle fatigue does not seem to elicit major detrimental changes to these jump-landing biomechanics. Although a wide variability of trunk and pelvic jump-landing strategies was observed, the results provide evidence for increased trunk flexion after lower extremity muscle fatigue. This proximal strategy is suggested to help unload fatigued lower extremity structures and lack of this compensation might increase knee injury risk.

## Introduction

Fatigue has recently been considered a candidate risk factor for sports injuries since a high proportion of these injuries occur during late game stages (Ekstrand et al., 2011; Gabbett, 2000; Hawkins et al., 2001; Price et al., 2004). Fatigue is a complex interaction between central and peripheral factors, and is, therefore, often broadly interpreted with different types of fatigue being defined in function of the studied mechanisms in literature (Verschueren et al., 2019). As such, one way to define fatigue is as the reduced capability of the neuromuscular system to react properly to incoming somatosensory information and biomechanical demands of a rapidly changing physical environment (Benjaminse et al., 2019; Enoka and Duchateau, 2008; MacIntosh and Rassier, 2002; Weinhandl et al., 2011). It is assumed that fatigue negatively affects other risk factors as it is associated with reduced muscle force, impaired neuromuscular control, suboptimal coordination, reduced postural control and impaired ankle joint position sense (Augustsson et al., 2006; Chappell et al., 2005; Kofotolis et al., 2007; Verschueren et al., 2019). However, the underlying mechanism for fatigue influencing injury risk is still unclear and the scientific debate whether fatigue is a risk factor or a modifying variable of risk factors remains inconclusive (Verschueren et al., 2019).

Since jump-landing tasks demand a high effort of the musculoskeletal system, a multitude of research has been performed to investigate the association between impaired jump-landing biomechanics and lower extremity injuries (De Bleecker et al., 2020; Hewett et al., 2005). In an attempt to better understand the underlying mechanism of fatigue in this context, previous systematic reviews have focused on the effect of fatigue on lower extremity jump-landing biomechanics in view of lower extremity injury risk (Barber-Westin and Noyes, 2017; Benjaminse et al., 2019; Jayalath et al., 2018; Santamaria and Webster, 2010). Inconsistent results were reported regarding hip, knee and ankle jump-landing biomechanics with some studies highlighting a stiffer landing strategy and others demonstrating a more flexed pattern at these joints after fatigue. Despite the high heterogeneity observed in these studies, proximal structures such as trunk and pelvis were ignored as these segments were often not the primary focus of investigation.

The positioning of proximal structures during jump-landing tasks seems to, however, play an important role in lower extremity loading and injury risk (Kibler et al., 2006). Increased trunk flexion during landing is associated with reduced ground reaction forces, whereas side-bending of the trunk leads to a lateral displacement of the ground reaction force and consequent increased knee abduction loads (Blackburn and Padua, 2008; Hewett et al., 2009; Shimokochi et al., 2013). Furthermore, adequate activity of the lumbo-pelvic-hip muscles helps maintain trunk stability and controls lower extremity movement during functional activities (Mirzaie et al., 2019). As such, the neuromuscular control of proximal structures plays an important role in the development and prevention of lower extremity injuries during jump-landing tasks (Chuter and Janse de Jonge, 2012; De Blaiser et al., 2018; De Bleecker et al., 2020). Although fatigue is associated with impaired neuromuscular control (Augustsson et al., 2006; Chappell et al., 2005; Kofotolis et al., 2007), it is still unclear whether trunk and pelvic movement strategies during jump-landing tasks are altered in a fatigued state.

Identifying fatigue-related biomechanical and neuromuscular changes of the trunk and pelvis during jump-landing tasks may provide new insight into the role of fatigue as a risk factor for injury development (Benjaminse et al., 2019; Santamaria et al., 2010). Therefore, the aim of this systematic review was to gather and synthesize the available evidence for the effect of fatigue on trunk and pelvic biomechanics and muscular activity during jump-landing tasks in healthy, physically active populations.

## Methods

### Eligibility Criteria

Studies reporting the effect of acute physical fatigue on trunk and/or pelvic kinematics, kinetics and/or muscular activity measured by electromyography during a landing from double-leg (DL) and single-leg (SL) jumps and jump-landing related cutting tasks in healthy, physically active populations were included (Table 1).

**Table 1:** Eligibility criteria.

	Inclusion	Exclusion
**P**	Physically active subjects (regular participation in sports during leisure time, work or education programs) Age ≥ 18y Healthy	Physically inactive or physical activity not reported Age < 18y Recent acute/overuse injuries ≤ 6 months Surgery of the lower extremity Other neurological or system diseases
**I**	Local and/or general exercise-induced fatigue protocol	Mental fatigue/dual tasks or other interventions (e.g., modifying landing kinematics via feedback)
**C**	Pre- vs. post-fatigue	Patient vs. control
**O**	Trunk and pelvic jump-landing kinematics and kinetics, assessed post-fatigue and measured by video (2D) and/or motion capture (3D) analysis DL and/or SL jump-landings and jump-landing related cutting tasks Muscular activity of trunk and/or pelvic muscles (ESL, EST, TrA, EO, IO, RA, GMax, GMed, TFL), measured by EMG	Subjective assessment of trunk and pelvic jump-landing kinematics, assessed post-fatigue and measured by clinical tests such as the LESS Running-related cutting maneuvers Lower extremity (hip, knee and/or ankle) jump-landing kinematics and kinetics Kinematics and kinetics during push-off Muscular activity of lower extremity muscles such as ST, SM, BF, RF, ADD musculature, measured by EMG

ADD = Adductor; BF = Biceps femoris; DL = Double-legged; EMG = Electromyography; EO = External oblique; ESL = Erector spinae, pars lumbalis; EST = Erector spinae, pars thoracalis; GMax = Gluteus maximus; GMed = Gluteus medius; IO = Internal oblique; LESS = Landing Error Scoring System; RA = Rectus abdominis; RF = Rectus femoris; SL = Single-legged; SM = Semimembranosus; ST = Semitendinosus; TFL = Tensor fascia latae; TrA = Transverse abdominis; 2D = two-dimensional; 3D = three-dimensional.

### Search Strategy and Information Sources

This systematic review was registered in the PROSPERO international prospective register of systematic reviews (ID=CRD42020154706) and conducted following the Preferred Reporting Items for Systematic reviews and Meta-analyses (PRISMA) 2020 guidelines (Appendix A1) (Ardern et al., 2022; Page et al., 2021). A PICO-model was designed to answer the research question: “What is the effect of fatigue (I) on trunk and pelvic jump-landing biomechanics (O) in healthy, physically active populations (P)?” No control group (C) was determined. The updated PROSPERO protocol can be accessed via: https://www.crd.york.ac.uk/prospero/display_record.php?RecordID=154706

The following electronic databases: PubMed (MEDLINE), Web of Science, Embase, CINAHL, and SPORTDiscus, were consulted from their inception until the 20^th^ of April 2022. Free text words, search terms, and corresponding MeSH terms (for PubMed) and EMTREE terms (for Embase) were combined with Boolean operators to answer the research question (Appendix A2). Furthermore, the reference lists of included studies were hand-searched afterwards to identify other relevant articles.

### Selection Process

Two authors screened independently for potential studies on the title and the abstract. The full texts of articles which remained after this first screening were retrieved and evaluated by the same authors. Discrepancies between the two authors were resolved with a consensus meeting. A third author was consulted in the event of disagreement. Inter-rater agreement for screening was calculated (IBM SPSS statistics 26) and expressed as the percentage of agreement (PoA) and k-statistics (De Bleecker et al., 2020).

### Data Collection Process, Data Items, Summary Measures and Synthesis Methods

An evidence table was made after collecting and summarizing the main study results (Table 2). This process was again performed independently by two authors. Studies were clustered per type of the fatigue protocol (general, local or task-specific) and for each study the following topics were reported: reference of the study (1), participants’ characteristics (2), fatigue protocol (3), landing task (4), outcome measures (5), and fatigue effects (6). For each study, changes in trunk and/or pelvic jump-landing biomechanics before and after fatigue were presented with the level of significance set at *p* < 0.05, if sufficient data were available from the original publication.

**Table 2:** Summary of Evidence. **Table 2a**. General fatigue protocols.

Study	Participants’ characteristics	Fatigue protocol	Landing task	Outcome measures	Fatigue effects
Abergel et al. (2020)	21♀ dancers with ≥5y experience (age: 19.6 ± 5.7y)	Dance-specific fatigue choreography (common jumps, leaps, and turns) until BORG-20 ≥ 17	DL sauté (n=12)	3D pelvic kinematics (RoM from IC to peak angle), immediately post-fatigue	**Pelvic kinematics**: *↑ anterior pelvic tilt (*p* < 0.001)
Kim et al. (2015)	21 (14♂, 7♀) physically active subjects (≥1.5h/week, ≥3x0.5h; age: 23.0 ± 3.0y)	3 exercises (5 min incremental running, 20 s lateral CMJ with a cadence controlled by a metronome (88 Hz), 20 max alternating vertical CMJ from lunging position, 10 s between exercises) until BORG-20 ≥ 17 and max vertical jump height ↓ with ≥20%	SL forward (1 m distance) side-cutting on dominant leg (n=5)	sEMG of GMax on dominant leg (whole curve analysis during ground contact phase), immediately post- fatigue	**sEMG of GMax**: *↓ activity 0–5% and at 35% of ground contact phase (*p* < 0.05)
Lessi et al. (2017)	40 recreational athletes (≥3x/week; 20♂, age: 22.8 ± 2.9y; 20♀, age: 23.6 ± 3.0y)	3 exercises (10 DL squats until 90° knee FLEX, 2 max vertical jumps, 20 step-ups onto 31 cm heights) until average distance during 3 max SL hops ↓ with ≥20%	SL DVJ on dominant leg from 31 cm box (n=3)	3D trunk and pelvic kinematics (angles at IC and peak), and sEMG of GMed and GMax (average amplitude from IC to peak knee FLEX), immediately post- fatigue	**Trunk kinematics**: no diff for FLEX at IC (*p* > 0.05), *↑ FLEX at peak (*p* < 0.001), no diff for SB at IC and peak (*p* > 0.05) **Pelvic kinematics**: *↑ contralat. pelvic drop at IC and peak (*p* < 0.001) **sEMG of GMed**: no diff (*p* > 0.05) **sEMG of GMax**: *↑ activity (*p* = 0.013)
Liederbach et al. (2014)	Group 1: 40 ballet/modern dancers (20♂, age: 27.0 ± 6.0y; 20♀, age: 25.0 ± 5.0y)	Circuits of 2 exercises (50 step-ups onto 30 cm heights, 15 SL max vertical jumps) until max SL vertical jump height ↓ with ≥10%	SL DVJ on dominant leg from height of 30 cm (n=3)	3D trunk kinematics (peak angles), not specified at which moment this was assessed post- fatigue	**Trunk kinematics**: *↑ FLEX (*p* = 0.002) and *↑ (right) SB (*p* < 0.001) in group 1 and 2
	Group 2: 40 athletes from jumping/cutting sports (20♂, age: 22.0 ± 2.0y; 20♀, age: 20.0 ± 2.0y)				
Smeets et al. (2019)	18 (10♂, 8♀) competitive athletes (≥3x/week; age: 21.3 ± 1.5y)	SAFT-5: 5 min soccer match simulation (sprinting, jogging, agility drills, slalom, CMJ and scissor jumps)	SL jumps (n=3): hop for distance (n=3), medial hop (n=3), vertical hop with 90° of med ROT (n=3)	sEMG of GMed bilateral (whole curve analysis from IC to 500 ms after IC), not specified at which moment this was assessed post- fatigue	**sEMG of GMed**: no diff (*p* > 0.05)
Smeets et al. (2020)	21 (15♂, 6♀) competitive athletes (training ≥2x/week; match ≥1x/week; age: 21.5 ± 1.5y): soccer (n=13), volleyball (n=4), basketball (n=2), hurdles (n=1), and dancing (n=1)	SAFT-5 (5 min)	SL max vertical hop with 90° of medial ROT on dominant and non-dominant leg (n=3)	3D trunk on pelvic kinematics (whole curve analysis from IC to 500 ms after IC), not specified at which moment this was assessed post-fatigue	**Trunk on pelvic kinematics**: *↓ FLEX during 13–179 ms of the vertical hop with medial ROT (*p* < 0.001, small ES)
Whyte et al. (2018a)	22♂ university athletes (≥3x/week; age: 21.9 ± 1.1y)	HIIP (circuits of forward/backward sprints (5 m), 10 DL forward jumps over 30 cm hurdles, 10 side- stepping exercises over 30 cm hurdles, and 4 side shuffles (5 m), 30 s rest between circuits) until BORG-20 ≥ 18	DL DVJ from height of 30 cm (n=3)	3D trunk on pelvic kinematics (whole curve analysis from IC to 1^st^ occurrence of concentric centre of mass power), immediately post- fatigue	**Trunk on pelvic kinematics**: *↑ FLEX during whole landing phase (*p* < 0.001, medium ES)
Whyte et al. (2018b)	28♂ university Gaelic football athletes (≥3x/week; age: 21.7 ± 2.2y)	HIIP until BORG-20 ≥ 18	SL (un)anticipated crossover cutting (45°): forward jumps (70% max jump distance) on dominant leg	3D trunk, trunk on pelvic and pelvic kinematics (whole curve analysis from IC to 1^st^ minimum vGRF), 30 s post- fatigue	**Trunk kinematics**: no diff for FLEX, SB and ROT (*p* > 0.05) **Trunk on pelvic kinematics**: no diff for FLEX, SB and ROT (*p* > 0.05)
					**Pelvic kinematics**: *↓ anterior pelvic tilt 84–100% of landing phase (*p* = 0.049; small ES), no diff for pelvic drop and ROT (*p* > 0.05)
Whyte et al. (2018c)	28♂ university Gaelic football athletes (≥3x/week; age: 21.7 ± 2.2y)	HIIP until BORG-20 ≥ 18	SL (un)anticipated side-cutting (45°): forward jumps (70% max jump distance) on dominant leg	3D trunk, trunk on pelvic and pelvic kinematics (whole curve analysis from IC to 1^st^ minimum vGRF), not specified at which moment this was assessed post- fatigue	**Trunk kinematics**: *↑ FLEX 1–100% of landing phase (*p* < 0.001, small ES), *↑ SB away from cutting direction 1–88% of landing phase (*p* = 0.038, small ES), no diff for ROT (*p* > 0.05)
					**Trunk on pelvis kinematics**: *↑ SB away from cutting direction 1– 75% of landing phase (*p* = 0.039, small ES), no diff for FLEX and ROT (*p* > 0.05)
					**Pelvic kinematics**: *↓ anterior pelvic tilt 81–100% of landing phase (*p* = 0.049, small ES), no diff for SB and ROT (*p* > 0.05)
Wong et al. (2020)	12♀ college athletes (≥4–6x/week; age: 21.3 ± 1.5 y): volleyball (n=4) and basketball (n=8)	Circuits of 2 exercises (50 step- ups onto 30 cm heights, 15 SL max vertical jumps) until max vertical jump height ↓ with ≥10% and BORG-20 ≥ 17	DL DVJ from 30 cm box positioned at 50% of subjects’ height (n=5)	3D trunk on pelvic kinematics (angles at IC), not specified at which moment this was assessed post- fatigue	**Trunk on pelvic kinematics**: *↓ FLEX at IC (*p* = 0.001)

**Table 2b:** Local fatigue protocols.

Study	Participants’ characteristics	Fatigue protocol	Landing task	Outcome measures	Fatigue effects
Becker et al. (2017)	12 amateur soccer players (not goalkeepers; age: 23.6 ± 4.2y)	3 abdominal and 2 dorsal exercises (abdominal: dynamic leg raising, sit-ups with dynamic rolling off, static forearm push- ups; dorsal: static and dynamic trunk extension; 60 s rest between sets) until subjective complete fatigue	DL landing after a header (n=3)	sEMG of EST, ESL 0.5 s after ball contact (iEMG), 60 s post- fatigue	**sEMG of EST**: *↓ activity (*p* = 0.015; medium ES) **sEMG of ESL**: no diff (*p* > 0.05)
Gafner et al. (2018)	19 (11♂, 8♀) recreationally active subjects (age: 30.3 ± 4.0y)	2 sets of repeated side-lying 30° hip ABD for dominant leg at 60 bpm (30 s rest between sets) until inability of performing 2 consecutive repetitions of the fatigue protocol	SL forward jump (25 cm distance) on dominant leg (n=1)	sEMG of GMed (onset time, average EMG at IC, peak and average EMG from IC to 250 ms after IC), 2 min post- fatigue	**sEMG of GMed**: no diff in onset time and activity at IC and from IC to 250 ms after IC (*p* > 0.05)
Hollman et al. (2012)	20♀ moderately and highly physically active subjects (IPAQ ≥600/week; age: 22.9 ± 1.8y)	Group 1 (experimental): modified Biering-Sørenson (prone isometric hip extension with unsupported trunk) until subjective fatigue	DL max vertical jump (n=3)	sEMG of GMax (peak EMG from IC to peak knee FLEX), 90 s post- fatigue	**sEMG of GMax**: Group 1: *↑ activity (*p* = 0.031) Group 2: no diff in GMax activity (*p* > 0.05)
		Group 2 (sham group): push-ups until subjective fatigue			
Kim et al. (2021)	10 (5♂, 5♀) physically active subjects (age: 26.6 ± 1.4y)	3 sets of side-lying 35° hip ABD at 60 bpm (2 min rest between sets) until inability to reach 35° hip ABD and confirmation through sEMG GMed	SL drop landing on dominant leg from 45 cm box (n=3)	3D trunk kinematics (peak angles and RoM from IC until peak knee FLEX), 3D trunk kinetics (whole curve analysis from IC until peak knee FLEX), immediately post-fatigue (within 1 min)	**Trunk kinematics**: *↑ SB at peak (*p* < 0.001; medium ES) and *↑ SB RoM (*p* = 0.009. medium ES) towards dominant leg
					**Trunk kinetics**: no diff in trunk extension, SB and ROT moment (*p* > 0.05)
Lin et al. (2021)	12♂ volleyball players (1^st^ division; 9.8 ± 1.7y experience; age: 19.0 ± 0.8y)	3 sets of raising shoulders in supine position ± 10 cm, 50 times at 45 bpm (30 s rest between sets) until full completion of protocol and confirmed through sEMG RA	DL CMJ and spike jump (n=3)	sEMG of RA and erector spinae on dominant (braking) leg and non- dominant (jumping) leg, not specified at which moment this was assessed post-fatigue	**sEMG of RA** on dominant and non-dominant leg: no diff (*p* > 0.05) **sEMG of erector spinae** on dominant and non-dominant leg: no diff (*p* > 0.05)
Patrek et al. (2011)	20♀ physically active subjects (≥1.5h/week, ≥3x0.5h; age: 21.0 ± 1.3y)	Repeated side-lying 30° hip ABD at 60 bpm until BORG-20 ≥ 19 and inability of performing 2 consecutive repetitions of fatigue protocol	SL drop landings on dominant leg from a 40 cm hang bar (n=5)	3D pelvic kinematics (angles at IC and 60 ms after IC), sEMG of GMed (onset time, peak EMG and iEMG during 1^st^ 60 ms after IC), 60 s post-fatigue	**Pelvic kinematics**: no diff for pelvic drop at IC and 60 ms after IC (*p* > 0.05) **sEMG of GMed**: *↑ onset time (*p* < 0.001; medium ES), no diff for peak EMG and iEMG (*p* > 0.05)
Rabello et al. (2021)	17 (8♂, 9♀) subjects participating in strength training programs (6.1 ± 4.2 y experience; age: 28.4 ± 6.1y)	Repeated side-lying hip ABD at 60 bpm (completion of 4 sets until concentric fatigue at 10 RM load, 2 min rest between sets)	SL hop for distance on dominant leg (n=3)	sEMG of GMed and TFL on dominant leg (peak values during eccentric phase), 2 min post- fatigue	**sEMG of GMed and TFL**: no diff (*p* > 0.05)

**Table 2c:** Task-specific fatigue protocols.

Study	Participants’ characteristics	Fatigue protocol	Landing task	Outcome measures	Fatigue effects
Matsunaga et al. (2021)	9♂ recreationally active subjects (2– 3x/week; age: 20.8 ± 2.2y): soccer, badminton, baseball, and rugby	Repeated side-jumps with a distance of 1.1x subject’s height until subjects were unable to jump in synchronicity with the metronome (60 Hz) or to jump the distance of the subject’s height	DL side-jump with a distance of 1.1x subject’s height (n=5)	sEMG of the right RA, EO, IO/TrA, ESL, GMed (mean frequencies, 200 ms before landing until 200 ms after TO), not specified at which moment this was assessed post-fatigue	**sEMG of RA, EO, IO/TrA, ESL, GMed**: no diff (*p* > 0.05)
McNeal et al. (2010)	20 1^st^ division athletes (11♂ runners, age: 21.4 ± 1.6y; 9♀ jumpers and throwers, age: 21.9 ± 3.7y)	60 s repeated DL max CMJ	DL max CMJ with landings up to 90° knee FLEX (n=3)	2D trunk kinematics (angles at 90° knee FLEX), assessed at 10 s intervals from the initial jump to the final 10 s during fatigue protocol	**Trunk kinematics**: *↑ FLEX (*p* < 0.001)
Weinhandl et al. (2011)	12 recreationally active university students (≥3x/week; 6♂, age: 22.0 ± 2.0y; 6♀, age: 22.0 ± 1.0y)	Repeated max DL DVJ every 20 s until mean DL DVJ height ↓ with ≥20% for 3 consecutive repetitions	DL DVJ from height of 20 cm	3D trunk kinematics (angles at IC and RoM from IC to peak FLEX during 1^st^ 100 ms after IC), assessed between initial 10% and final 10% jumps during fatigue protocol	**Trunk kinematics**: no diff for FLEX at IC (*p* > 0.05) and *↑ FLEX RoM (*p* = 0.007; large ES)
Wild et al. (2017)	14♀ Irish dancers (age: 19.4 ± 3.7y)	Repeated leap over until BORG-20 ≥ 17 and ↓ performance	SL leap over with landing on the right leg (n=5)	3D trunk kinematics (peak angles from IC to TO), immediately post-fatigue	**Trunk kinematics**: no diff for FLEX and SB (*p* > 0.05)

* Main results: Differences between pre- and post-fatigue are presented with the significant level set at p < 0.05 (if sufficient data were available from the original publication).

ABD = Abduction; Bpm = Beats per minute; Contralat. = Contralateral; CMJ = Countermovement jumps; Diff = Difference; DL = Double-leg; DVJ = Drop vertical jump; EO = External oblique; ES = Effect size; ESL = Erector spinae, pars lumbalis; EST = Erector spinae, pars thoracalis; FLEX = Flexion; GMax = Gluteus maximus; GMed = Gluteus medius; HIIP = High intensity, intermittent exercise protocol; iEMG = integrated electromyography; IC = Initial contact; IO = Internal oblique; Max = Maximal; min = minute(s); ms = milliseconds; RA = Rectus abdominis; RoM = Range of motion; RM = Repetition maximum; ROT = Rotation; s = second(s); SAFT = Soccer-specific aerobic field test; SB = Side-bending; sEMG = surface electromyography; SL = Single-leg; TFL = Tensor fascia latae; TO = Take-off; TrA = Transversus abdominis; vGRF = vertical ground reaction force.

**Figure 1:**
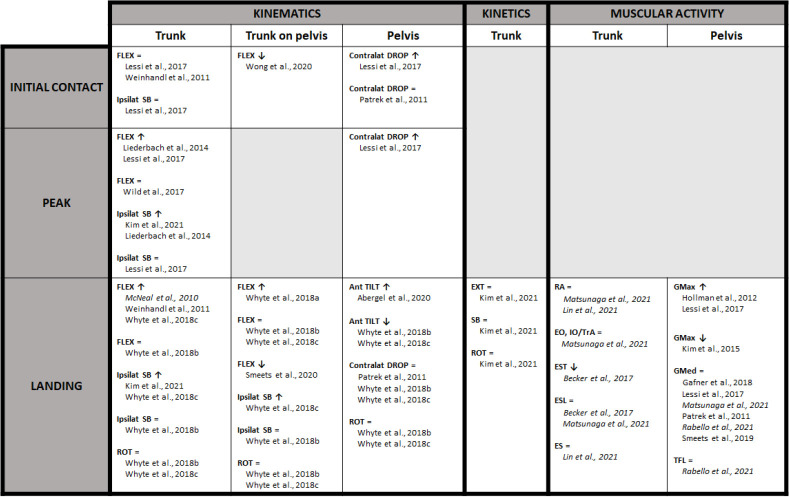
Trunk and Pelvic Landing Biomechanics of Double- and Single-leg Jumps and Jump-landing Related Cutting Tasks after Fatigue. Studies with high methodological quality are written in normal format, those with moderate methodological quality are highlighted in italics. *Ant TILT = Anterior tilt; Contralat DROP = Contralateral drop; EO = External oblique; ES = Erector spinae; ESL = Erector spinae, pars lumbalis; EST = Erector spinae, pars thoracalis; EXT = Extension; FLEX = Flexion; GMax = Gluteus maximus; GMed = Gluteus medius; IO = Internal oblique; Ipsilat SB = Ipsilateral side-bending; RA = Rectus abdominis; ROT = Rotation; TFL = Tensor fascia latae; TrA = Transversus abdominis*.

The evidence for changes in trunk and/or pelvic biomechanics during DL or SL landings, and/or jump-landing related cutting tasks after fatigue was clustered per the anatomical region (Figure 1). Trunk kinematics were considered as the absolute movement of the trunk relative to the global coordinate system. Trunk on pelvic kinematics were considered as movement of the trunk relative to the pelvis. Pelvic kinematics represented the absolute movement of the pelvic segment relative to the global coordinate system. Fatigue-related changes in kinematics, in the form of the range of motion for the above mentioned segments, were subtracted at initial contact, during the entire landing phase and at kinematic peak angles. Furthermore, trunk and/or pelvic joint moments after fatigue were presented as kinetic variables. Finally, changes in trunk and/or pelvic muscular activity after fatigue were presented per muscle group.

### Quality Assessment

The methodological quality of each study was evaluated independently by two authors using a modified version of the Downs and Black checklist (Appendix A3) (Downs and Black, 1998). This version had previously been used in similar systematic reviews (Benjaminse et al., 2019; De Bleecker et al., 2020; Santamaria et al., 2010). Fifteen items (items 1, 2, 3, 4, 5, 6, 7, 8, 10, 11, 12, 16, 18, 20, 25) with a combined maximum result of 16 points were scored for each study. The items could be rewarded with a maximum score of 1 point (1 = “yes”, 0 = “no”, 0 = “not able to determine”). Only the fifth checklist item could be rewarded with a maximum score of 2 points (2 = “yes”, 1 = “partially”, 0 = “no”). For the included studies, a score of ≥ 11 was considered as high quality, 6–10 was considered as moderate quality, and ≤ 5 was considered as low quality (Neal et al., 2016). A third author was consulted in the event of disagreement. PoA and k-statistics were calculated to check inter-rater agreement.

Based on methodological quality and the applied design, each included study received a level of evidence according to the 2005 classification system of the Dutch Institute for Healthcare Improvement CBO (Meeus and Gebruers, 2016). Finally, a level of conclusion was made after clustering results of studies with comparable methodological quality. The levels of conclusion ranged from 1 to 4 and corresponded to a high (1), moderate (2), low strength of conclusion (3) or no strength of conclusion at all (4) (Meeus and Gebruers, 2016).

## Results

### Study Selection

**Figure 2:**
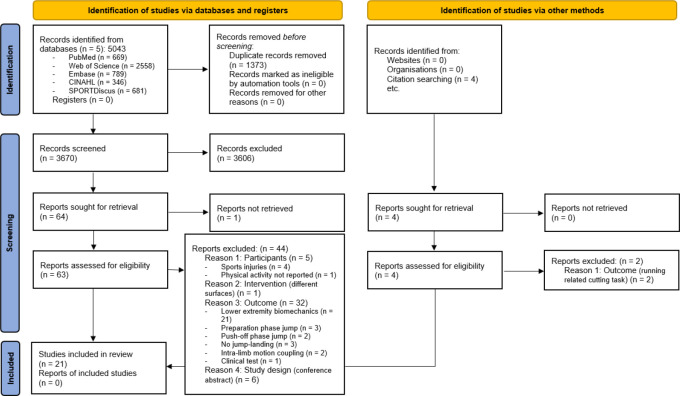
Flow Diagram of the Study Selection Process. From: Page MJ, McKenzie JE, Bossuyt PM, Boutron I, Hoffmann TC, Mulrow CD, et al. The PRISMA 2020 statement: an updated guideline for reporting systematic reviews. BMJ 2021;372:n71. doi: 10.1136/bmj.n71. For more information, visit: http://www.prisma-statement.org/

The electronic databases search yielded a total of 5043 citations. More specifically, 669 articles were retrieved from PubMed (MEDLINE), 2558 from Web of Science, 789 from Embase, 346 from CINAHL and 681 from SPORTDiscus. After removing duplicates, 3670 articles were screened. Screening on the title and the abstract resulted in 63 studies (PoA = 97.5%, k-score = 65.1%; *p* < 0.001), of which 21 articles fulfilled the eligibility criteria after full-text screening and hand-searching (PoA = 87.9%, k-score = 73.4%; *p* < 0.001) (Figure 2).

### Study Characteristics

#### 1. Participants’ Characteristics

A total of 428 subjects (211 males, 205 females, 12 not mentioned) participated in the included studies. Seven studies evaluated landing biomechanics in athletes performing repetitive jumping and jump-landing related cutting tasks such as volleyball and basketball (Becker et al., 2017; Lin et al., 2021; McNeal et al., 2010; Smeets et al., 2020; Whyte et al., 2018b, 2018c; Wong et al., 2020). Four studies included a ballet/dance population (Abergel et al., 2020; Liederbach et al., 2014; Smeets et al., 2020; Wild et al., 2017). Three studies examined university athletes (Whyte et al., 2018a, 2018b, 2018c), and one study evaluated athletes performing strength training programs (Rabello et al., 2021). Finally, nine studies investigated recreational athletes (Gafner et al., 2018; Hollman et al., 2012; Kim et al., 2015, 2021; Lessi et al., 2018; Matsunaga et al., 2021; Patrek et al., 2011; Smeets et al., 2019; Weinhandl et al., 2011).

#### 2. Jump-Landing Task Characteristics

A variety of jump-landing tasks with different levels of complexity were evaluated across the included studies. Nine studies investigated biomechanics during DL landings (Abergel et al., 2020; Becker et al., 2017; Hollman et al., 2012; Lin et al., 2021; Matsunaga et al., 2021; McNeal et al., 2010; Weinhandl et al., 2011; Whyte et al., 2018a; Wong et al., 2020), and twelve studies used SL landings (Gafner et al., 2018; Kim et al., 2015, 2021; Lessi et al., 2017; Liederbach et al., 2014; Patrek et al., 2011; Rabello et al., 2021; Smeets et al., 2019, 2020; Whyte et al., 2018b, 2018c; Wild et al., 2017).

Fifteen studies investigated biomechanics during standardized jump-landing tasks (Gafner et al., 2018; Hollman et al., 2012; Kim et al., 2021; Lessi et al., 2017; Liederbach et al., 2014; Lin et al., 2021; Matsunaga et al., 2021; McNeal et al., 2010; Patrek et al., 2011; Rabello et al., 2021; Smeets et al., 2019, 2020; Weinhandl et al., 2011; Whyte et al., 2018a; Wong et al., 2020), whereas seven studies used sports-specific jump-landing tasks (Abergel et al., 2020; Becker et al., 2017; Kim et al., 2015; Lin et al., 2021; Whyte et al., 2018b, 2018c; Wild et al., 2017). With regard to the sports-specific jump-landing tasks, three studies investigated jump-landing related crossover cutting or side-cutting tasks (Kim et al., 2015; Whyte et al., 2018b, 2018c), two studies dance-specific jump-landing tasks (Abergel et al., 2020; Wild et al., 2017), one study soccer-specific jump-landing tasks (Becker et al., 2017), and one study volleyball-specific jump-landing tasks (Lin et al., 2021).

#### 3. Fatigue Protocol Characteristics

Seven studies implemented a local fatigue protocol of the lumbo-pelvic-hip muscles (Becker et al., 2017; Gafner et al., 2018; Hollman et al., 2012; Kim et al., 2021; Lin et al., 2021; Patrek et al., 2011; Rabello et al., 2021). In these studies, fatigue was induced in the gluteus medius (Gafner et al., 2018; Kim et al., 2021; Patrek et al., 2011; Rabello et al., 2021), the gluteus maximus (Hollman et al., 2012), the erector spinae (Becker et al., 2017; Hollman et al., 2012), or the abdominal muscles (Becker et al., 2017; Lin et al., 2021).

Ten studies used a general fatigue protocol (Abergel et al., 2020; Kim et al., 2015; Lessi et al., 2017; Liederbach et al., 2014; Smeets et al., 2019, 2020; Whyte et al., 2018a, 2018b, 2018c; Wong et al., 2020). These protocols consisted of repeated functional circuits, with a fixed time (Smeets et al., 2019, 2020) or fixed subjective or objective measure of fatigue (Abergel et al., 2020; Kim et al., 2015; Lessi et al., 2017; Liederbach et al., 2014; Whyte et al., 2018a, 2018b, 2018c; Wong et al., 2020).

Finally, four studies used a task-specific fatigue protocol (Matsunaga et al., 2021; McNeal et al., 2010; Weinhandl et al., 2011; Wild et al., 2017). In these protocols, jump-landing biomechanics were extracted before and after a fixed-time repeated jump maneuver (McNeal et al., 2010), or fixed subjective or objective measure of fatigue (Matsunaga et al., 2021; Weinhandl et al., 2011; Wild et al., 2017). The task-specific fatigue protocols induced local lower extremity muscle fatigue combined with general fatigue.

### Quality Assessment

The modified Downs and Black checklist scores ranged from 9 to 14 out of 16 (PoA = 90.7%, k-score = 76.5%; *p* < 0.001). Sixteen studies received a high quality score (Abergel et al., 2020; Gafner et al., 2018; Hollman et al., 2012; Kim et al., 2015, 2021; Lessi et al., 2017; Liederbach et al., 2014; Patrek et al., 2011; Smeets et al., 2019, 2020; Weinhandl et al., 2011; Whyte et al., 2018a, 2018b, 2018c; Wild et al., 2017; Wong et al., 2020), whereas five studies were of moderate quality (Becker et al., 2017; Lin et al., 2021; Matsunaga et al., 2021; McNeal et al., 2010; Rabello et al., 2021). Average study quality was 71.4%, corresponding to an average high study quality. All the included studies received a level of evidence B (Table 3).

**Table 3:** Individual study quality and Corresponding Level of Evidence of the Individual Studies.

Study	1	2	3	4	5	6	7	8	10	11	12	16	18	20	25	Total	LoE
Abergel et al. (2020)	1	0	1	1	0	1	1	1	1	1	0	1	1	1	0	11/16	B
*Becker et al. (2017)*	*1*	*1*	*1*	*0*	*1*	*0*	*1*	*0*	*1*	*1*	*0*	*1*	*1*	*0*	*0*	*9/16*	*B*
Gafner et al. (2018)	1	1	1	1	1	1	1	0	1	1	0	1	1	1	0	12/16	B
Hollman et al. (2012)	1	1	1	0	1	1	1	0	1	1	0	1	1	1	1	12/16	B
Kim et al. (2015)	1	1	1	1	2	0	1	0	0	1	0	1	1	1	0	11/16	B
Kim et al. (2021)	1	1	1	1	1	1	1	0	1	1	0	1	1	1	0	12/16	B
Lessi et al. (2017)	1	1	1	1	2	1	1	0	1	1	0	1	1	1	1	14/16	B
Liederbach et al. (2014)	1	1	1	1	1	1	1	0	1	1	0	1	1	1	1	13/16	B
*Lin et al. (2021)*	*1*	*0*	*1*	*0*	*1*	*1*	*1*	*0*	*0*	*1*	*0*	*1*	*1*	*1*	*0*	*9/16*	*B*
*Matsunaga et al. (2021)*	*1*	*1*	*1*	*1*	*1*	*0*	*0*	*0*	*1*	*0*	*0*	*1*	*1*	*1*	*0*	*9/16*	*B*
*McNeal et al. (2010)*	*0*	*1*	*0*	*1*	*1*	*0*	*1*	*1*	*1*	*1*	*0*	*1*	*1*	*0*	*1*	*10/16*	*B*
Patrek et al. (2011)	1	1	1	1	2	1	1	0	1	1	0	1	1	1	0	13/16	B
*Rabello et al. (2021)*	*0*	*1*	*1*	*1*	*1*	*1*	*1*	*0*	*1*	*0*	*0*	*0*	*1*	*1*	*0*	*9/16*	*B*
Smeets et al. (2019)	1	0	1	1	2	1	1	0	1	1	0	1	1	1	0	12/16	B
Smeets et al. (2020)	1	0	1	1	2	0	1	0	1	1	0	1	1	1	1	12/16	B
Weinhandl et al. (2011)	0	1	1	1	2	1	1	0	1	0	0	1	1	1	0	11/16	B
Whyte et al. (2018a)	1	1	1	1	2	1	1	0	1	0	0	1	1	1	0	12/16	B
Whyte et al. (2018b)	1	1	1	1	2	1	1	0	1	1	0	1	1	1	0	13/16	B
Whyte et al. (2018c)	1	1	1	1	1	1	1	0	1	1	0	1	1	1	0	12/16	B
Wild et al. (2017)	1	0	1	1	2	1	1	0	1	1	0	1	1	1	0	12/16	B
Wong et al. (2020)	1	0	1	1	1	1	1	0	1	1	0	1	1	1	0	11/16	B

Studies with high methodological quality are written in normal format, those with moderate methodological quality are highlighted in italics.

LoE = Level of Evidence. 1) Clear aim, 2) outcomes described, 3) participants described, 4) interventions described, 5) principal confounders described, 6) main findings described, 7) estimates of random variability, 8) adverse events of the intervention described, 10) actual probability values reported, 11) participants representative for population, 12) subjects prepared to participate representative for population, 16) data dredging, 18) appropriate statistical tests, 20) accuracy of the main outcome measures, 25) adjustment for confounders.

### Synthesis of the Results

#### 1. Kinematics

##### 1.1. Trunk

At initial contact of DL and SL landings, two studies observed similar flexion angles before and after general fatigue (Lessi et al., 2017; Weinhandl et al., 2011). Furthermore, one study showed similar ipsilateral side-bending angles before and after general fatigue during SL landing at initial contact (Lessi et al., 2017).

During the landing phase of a DL landing, two studies demonstrated significantly more flexion (*p* = 0.001–0.007) after repeated DL landings until fatigue (McNeal et al., 2010; Weinhandl et al., 2011). One study found similar flexion and ipsilateral side-bending before and after general fatigue during the entire landing in SL crossover cutting (Whyte et al., 2018b). Another study reported significantly more flexion during the entire landing (*p* < 0.001) of SL side-cutting and significantly more side-bending away from the cutting direction from 1 to 88% of the landing phase (*p* = 0.038) after the same general fatigue protocol (Whyte et al., 2018c). Furthermore, one study demonstrated significantly more ipsilateral side-bending during a SL drop landing (*p* = 0.009) after local fatigue of the gluteus medius muscle (Kim et al., 2021). Similar rotation angles were observed in two studies before and after general fatigue when performing cutting tasks, regardless of cutting direction (Whyte et al., 2018b, 2018c).

Two studies showed significantly more peak trunk flexion during SL drop landings after general fatigue (*p* = 0.001–0.002) (Lessi et al., 2017; Liederbach et al., 2014). However, one study reported no differences for peak trunk flexion before and after repeated dance-specific SL landings until fatigue (Wild et al., 2017). There are inconsistencies for peak trunk ipsilateral side-bending during SL landings as one study demonstrated similar ipsilateral side-bending angles before and after general fatigue (Lessi et al., 2017), whereas two studies found significantly more ipsilateral side-bending (*p* < 0.001) after local fatigue of the gluteus medius muscle or general fatigue (Kim et al., 2021; Liederbach et al., 2014).

##### 1.2. Trunk on Pelvis

For trunk on pelvic kinematics, all studies examined joint angles after completion of a general fatigue protocol.

One study demonstrated significantly less flexion (*p* = 0.001) of the trunk relative to the pelvis after fatigue at initial contact of DL landings (Wong et al., 2020).

Trunk angles relative to the pelvis during landing were inconsistent after fatigue. One study showed significantly more flexion (*p* < 0.001) after fatigue during the entire landing phase of a DL drop jump (Whyte et al., 2018a). Another study demonstrated significantly less flexion (*p* < 0.001) after fatigue from 13 to 179 ms of a SL landing (Smeets et al., 2020). Two studies reported similar flexion and rotation when performing a SL cutting maneuver after fatigue, regardless of cutting direction (Whyte et al., 2018b, 2018c). Similar side-bending angles before and after fatigue during the entire landing of crossover cutting were demonstrated in one study (Whyte et al., 2018b), whereas significantly more side-bending away from cutting direction from 1 to 75% of the landing phase (*p* = 0.039) was observed after the same fatigue protocol in side-cutting in another study (Whyte et al., 2018c).

##### 1.3. Pelvis

Similar frontal plane pelvic kinematics were observed in one study before and after local fatigue of the gluteus medius muscle at initial contact during SL drop landings (Patrek et al., 2011), whereas another study found significantly more contralateral pelvic drop during SL landings (*p* < 0.001) after general fatigue (Lessi et al., 2017).

During the landing phase of SL jumps, one study demonstrated a significantly more anterior pelvic tilt (*p* < 0.001) after general fatigue during DL dance-specific sautés (Abergel et al., 2020). However, two studies found a less anterior pelvic tilt from 81 to 100% and 84 to 100% of the landing phase (*p* = 0.049) after general fatigue in SL cutting (Whyte et al., 2018b, 2018c). Three studies demonstrated similar contralateral pelvic drop before and after local fatigue of the gluteus medius muscle or general fatigue (Patrek et al., 2011; Whyte et al., 2018 b, 2018c). Finally, similar pelvic rotation angles were observed during the entire landing after general fatigue in two studies (Whyte et al., 2018b, 2018c).

Finally, one study showed significantly more peak contralateral pelvic drop (*p* < 0.001) after general fatigue during SL landings (Lessi et al., 2017).

#### 2. Kinetics

Only one study examined trunk kinetics before and after fatigue during a SL drop landing. Similar trunk extension, side-bending and rotation moments were observed before and after local fatigue of the gluteus medius muscle during the entire landing (Kim et al., 2021).

#### 3. Muscular Activity

##### 3.1. Trunk

One study demonstrated similar activity of the abdominal muscles (rectus abdominis, external and internal oblique, and transversus abdominis) during DL landing of a side-jump before and after repeated side-jumps until fatigue (Matsunaga et al., 2021). Two studies found similar muscular activity of the lumbar part of the erector spinae during DL landings before and after local fatigue of the lumbo-pelvic-hip muscles (Becker et al., 2017; Matsunaga et al., 2021). Furthermore, significantly lower muscular activity of the thoracic part of the erector spinae (*p* = 0.015) was observed in one study during DL landings before and after local fatigue of the erector spinae and abdominal muscles (Becker et al., 2017). Finally, one study demonstrated similar activity of the rectus abdominis and erector spinae during DL landings of standardized countermovement jumps and volleyball-specific spike jumps before and after local fatigue of the abdominal muscles (Lin et al., 2021).

##### 3.2. Pelvis

Two studies found significantly higher muscular activity of the gluteus maximus (*p* = 0.013–0.031) during DL and SL landings after local fatigue of the erector spinae and gluteus maximus muscles or general fatigue (Hollman et al., 2012; Lessi et al., 2017), whereas another study found significantly lower gluteus maximus muscular activity from 0 to 5% and at 35% of landing (*p* < 0.05) during a SL cutting task after general fatigue (Kim et al., 2015). Six studies showed similar activation levels for the gluteus medius muscle during the entire landing of a DL and/or SL jump before and after local fatigue of the gluteus medius muscle and/or general fatigue (Gafner et al., 2018; Lessi et al., 2017; Matsunaga et al., 2021; Patrek et al., 2011; Rabello et al., 2021; Smeets et al., 2019). Finally, one study demonstrated similar activation levels for the tensor fascia latae muscle during the entire landing of a SL hop for distance after local fatigue of the gluteus medius muscle (Rabello et al., 2021).

## Discussion

### Summary of Evidence

This is the first systematic review to summarize the current evidence regarding the effect of fatigue on trunk and pelvic biomechanics during various jump-landing tasks in healthy, physically active populations. In this systematic review, a high heterogeneity of fatigue protocols and jump-landing tasks across the different studies was found. Likewise, the review revealed a wide variety of trunk and pelvic landing strategy alterations after fatigue. Despite the heterogeneity in fatigue protocols and landing tasks, several general adaptive patterns can be distinguished. It seems that lower extremity muscle fatigue, induced by general or task-specific fatigue protocols, does indeed elicit biomechanical alterations in terms of an increased trunk flexion with more gluteus maximus muscular activity during standardized jump-landing tasks, although this is currently not clear for sports-specific jump-landing tasks. On the other hand, lumbo-pelvic-hip muscle fatigue, induced by local fatigue protocols, only seems to induce detrimental alterations in trunk kinematics in terms of increased ipsilateral side-bending, but does not appear to affect pelvic kinematics and lumbo-pelvic-hip muscular activations to a major extent.

Sagittal plane biomechanics after lower extremity muscle fatigue seems to be characterized by similar trunk flexion angles at initial contact, yet more trunk flexion during landing and at peak in standardized jump-landing tasks (conclusion level 2). Inconsistent results were found for post-fatigue trunk and pelvic kinematics during sports-specific tasks (level of conclusion 3). Additionally, fatigue-related changes in gluteus maximus muscular activity demonstrated also inconsistent results, with more activity during a standardized jump-landing task (level of conclusion 3) and less activity during a sports-specific cutting task (level of conclusion 3). With regard to the results of standardized jump-landing tasks, landing with more trunk flexion and more gluteus maximus muscular activity reflects a positive adaptive strategy in order to accommodate impact forces acting on lower extremity structures, certainly when fatigue-induced stiffer distal joint behaviors occur (De Bleecker et al., 2020; Powers, 2010). Trunk flexion might reduce external knee joint moments during landing, which has been suggested to be a consequence of a closer positioning of the ground reaction force vector with respect to the knee joint (Powers, 2010). It has been shown that trunk flexion landing patterns decrease patellar tendon and anterior cruciate ligament load, which is suggested to decrease knee injury risk (Dingenen et al., 2015; Scattone Silva et al., 2017; Shimokochi et al., 2013). Although low evidence for this statement, it is still possible that proximal strategies vary depending on structure-specific fatigability since jump-specific fatiguing exercises predominantly targeting the knee extensor muscles elicit trunk flexion during landing (Liederbach et al., 2014; Lessi et al., 2017), whereas no trunk alterations were observed after dance-specific protocols that predominantly induce fatigue in the calf musculature (Wild et al., 2017).

Inconsistent results were found for frontal plane trunk biomechanics after lower extremity muscle fatigue (level of conclusion 3). As such, it seems that dancers or athletes participating in cutting/jumping sports integrate more trunk ipsilateral side-bending during standardized landings when fatigued (Liederbach et al., 2014), compared to recreational athletes from different sports (Lessi et al., 2017). Sports-specific adaptations may contribute to these different landing strategies since some sports-specific movements require trunk side-bending during the flight phase, which could have an impact on the biomechanical variables observed during landing (Hinshaw et al., 2019). For sports-specific jump-landing related cutting tasks, the results are also inconsistent for trunk side-bending, depending on the cutting direction (Whyte et al., 2018b, 2018c). Ipsilateral trunk side-bending might be utilized to support the fatigued lower extremity muscles during the re-direction of the center of mass when performing the side-cutting task. Re-direction of the center of mass by ipsilateral trunk side-bending causes a lateral displacement of the resultant ground reaction force vector (Powers, 2010), which results in a higher knee abduction moment and an increased anterior cruciate ligament injury risk (Hewett et al., 2005; Weltin et al., 2015). Despite the large inconsistencies for the trunk biomechanics, frontal plane pelvic biomechanics were consistently characterized by similar contralateral pelvic drop and gluteus medius activity before and after lower extremity muscle fatigue during standardized and/or sports-specific landings (conclusion level 2), except at initial contact and at peak where more contralateral pelvic drop was demonstrated for standardized landings (conclusion level 3). However, there is only limited evidence for this statement and more research on this matter is needed since contralateral pelvic drop is usually associated with a valgus pattern of the lower extremity, potentially resulting in increased knee abduction loading and injury risk (Hewett et al., 2005; Willson et al., 2008).

This review demonstrated similar transversal plane movements at the trunk and pelvis during sports-specific jump-landing related cutting tasks before and after lower extremity muscle fatigue (conclusion level 3). Only one research group investigated this relationship during cutting tasks (Whyte et al., 2018b, 2018c), which makes it difficult to draw strong conclusions. Although there is little evidence for this statement, rotational control of the entire kinetic chain does not seem to be affected by fatigue, even in complex, reactive jump-landing related cutting tasks. Maintaining a transversal plane joint position during landing is needed to prevent additional tensile forces acting on lower extremity structures (Verrelst et al., 2014).

Finally, fatigue of the lumbo-pelvic-hip muscles was shown to only induce detrimental alterations in frontal plane trunk kinematics resulting in more ipsilateral side-bending (conclusion level 3). As mentioned above, this strategy potentially leads to higher knee abduction loads and increased anterior cruciate ligament injury risk (Hewett et al., 2005; Weltin et al., 2015). Since only one study investigated the effect of local lumbo-pelvic-hip muscle fatigue on frontal plane trunk biomechanics, it is difficult to make strong conclusions. No major alterations were found for pelvic kinematics and lumbo-pelvic-hip muscular activations during jump-landing tasks after local lumbo-pelvic-hip muscle fatigue (conclusion level 2), which would reflect an efficient strategy to maintain proximal pelvic control during landing. However, this statement has to be interpreted with caution since it has been suggested that full muscle strength recovery would occur within 2 to 4 minutes following a local fatigue protocol, which may explain why no fatigue effects were observed after protocol cessation even if they may have been present during the execution of the protocol (Salavati et al., 2007).

### Methodological Considerations and Research Implications

Some methodological considerations need to be taken into account when interpreting the results of this systematic review. A wide variety of fatigue protocols, types of jumps and analyzed outcomes were described across the included studies which makes it difficult to generalize and interpret results. Considerable heterogeneity exists in how fatigue was induced (local vs. general vs. task-specific) and measured (objective vs. subjective) across the studies, with fatigue being broadly interpreted (Verschueren et al., 2019). The majority of the included studies used a fatigue protocol without having information about the extent to which fatigue effects persisted after the protocol’s completion. Only two studies used a fixed-demand general fatigue protocol (Smeets et al., 2019, 2020), that has been proven to induce long-lasting decreases in knee extensor muscle strength for up to 30 min (Bossuyt et al., 2016). To gain more insight into the effect of match-play-induced fatigue in a particular sporting context, validated fatigue protocols with sports-specific characteristics are required.

Besides heterogeneity in fatigue protocols, different types of jump-landing tasks (DL vs. SL, standardized vs. sports-specific) were analyzed. The minority of the included studies observed fatigue-related alterations during sports-specific jump-landing tasks (Abergel et al., 2020; Becker et al., 2017; Kim et al., 2015; Lin et al., 2021; Whyte et al., 2018b, 2018c; Wild et al., 2017). A wide variety of tasks were used in these studies (e.g., cutting, dancing, heading, spiking), leading to different movement strategy adaptations to these jump-landing tasks when fatigued. Since a high amount of repetitive, high-impact jumps are utilized in typical jump-landing sports such as volleyball and basketball, future studies should investigate trunk and pelvic strategy accommodations in a fatigued state when performing jump-landing tasks specific for these sports (e.g., stop/spike jump, block jump).

Considering the analyzed outcomes, seven studies analyzed the entire landing phase with specialized statistical methods (Kim et al., 2015, 2021; Smeets et al., 2019, 2020; Whyte et al., 2018a, 2018b, 2018c), which are considered more appropriate due to avoiding focus bias adequately corrected for multiple comparisons (De Ridder et al., 2015; Pataky, 2010). However, the results of full curve analysis could not be directly compared to the studies with biomechanical data on discrete points.

Due to the large heterogeneity regarding fatigue protocols, analyzed jump-landing tasks and biomechanical outcomes of the included studies, performing a meta-analysis was not appropriate. However, this systematic review gives a broad overview of the effect of fatigue on trunk and pelvic jump-landing biomechanics in healthy, physically active populations, being an underrepresented topic in literature up to this day. Despite the mix of the included studies, this systematic review found evidence for more trunk flexion during standardized jump-landing tasks after lower extremity muscle fatigue, and this information can be implemented in future research studies.

### Clinical Implications

The methodological differences among the included studies make it difficult to give strong clinical recommendations. However, it seems that sagittal plane flexion landing patterns during standardized jump-landing tasks are important fatigue-related adaptations to accommodate for increased landing impact forces with stiffer distal joint behaviors. More specifically, this proximal strategy is suggested to help unload fatigued lower extremity structures and lack of this compensation might increase knee injury risk (Dingenen et al., 2015; Scattone Silva et al., 2017; Shimokochi et al., 2013). Although there is currently limited evidence for sports-specific jump-landing tasks, a wide variability of fatigue-related strategies seems to be utilized, depending on the specificity of the task. Since a lot of sports activities impose environmental constraints to the flexion pattern (e.g., the proximity of the net in volleyball or an opponent in many team sports), frontal and transversal plane movement adaptations due to fatigue are hypothesized to be of greater importance than what the research evidence shows. Although current evidence is lacking, more frontal and transversal plane movements in a fatigued state may be associated with and predictive for lower quadrant acute and/or overuse injuries (De Bleecker et al., 2020; Haddas et al., 2016; Lessi and Serrão, 2017; Willson et al., 2008). Further high quality prospective cohort studies are needed to infer causality between biomechanical alterations to the lumbo-pelvic region during jump-landing tasks in a fatigued state and the risk of developing lower extremity injuries.

## Conclusions

There is preliminary evidence for trunk and pelvic biomechanical adaptive strategies during landing after fatigue in order to reduce and/or realign impact forces acting on lower extremity structures. Flexion landing patterns during standardized jump-landing tasks are demonstrated at the trunk after lower extremity muscle fatigue in order to accommodate for increased landing impact forces with stiffer distal joint behaviors, which consequently decrease knee injury risk. For sports-specific jump-landing tasks, the results are currently inconsistent due to limited evidence. Otherwise, lumbo-pelvic-hip muscle fatigue does not seem to elicit major detrimental changes to these jump-landing biomechanics. Despite the large methodological heterogeneity across the included studies, this systematic review provides a broad overview of the current evidence regarding the effect of fatigue on trunk and pelvic jump-landing biomechanics.

## Figures and Tables

**Appendix A1:** PRISMA 2020 Checklist.

Section and Topic	Item #	Checklist item	Location where item is reported
**TITLE**			
Title	1	Identify the report as a systematic review.	1
**ABSTRACT**			
Abstract	2	See the PRISMA 2020 for Abstracts checklist.	1
**INTRODUCTION**			
Rationale	3	Describe the rationale for the review in the context of existing knowledge.	1–2
Objectives	4	Provide an explicit statement of the objective(s) or question(s) the review addresses.	2
**METHODS**			
Eligibility criteria	5	Specify the inclusion and exclusion criteria for the review and how studies were grouped for the syntheses.	2
Information sources	6	Specify all databases, registers, websites, organisations, reference lists and other sources searched or consulted to identify studies. Specify the date when each source was last searched or consulted.	2
Search strategy	7	Present the full search strategies for all databases, registers and websites, including any filters and limits used.	2
Selection process	8	Specify the methods used to decide whether a study met the inclusion criteria of the review, including how many reviewers screened each record and each report retrieved, whether they worked independently, and if applicable, details of automation tools used in the process.	2–3
Data collection process	9	Specify the methods used to collect data from reports, including how many reviewers collected data from each report, whether they worked independently, any processes for obtaining or confirming data from study investigators, and if applicable, details of automation tools used in the process.	3
Data items	10a	List and define all outcomes for which data were sought. Specify whether all results that were compatible with each outcome domain in each study were sought (e.g. for all measures, time points, analyses), and if not, the methods used to decide which results to collect.	3
	10b	List and define all other variables for which data were sought (e.g. participant and intervention characteristics, funding sources). Describe any assumptions made about any missing or unclear information.	3
Study risk of bias assessment	11	Specify the methods used to assess risk of bias in the included studies, including details of the tool(s) used, how many reviewers assessed each study and whether they worked independently, and if applicable, details of automation tools used in the process.	3
Effect measures	12	Specify for each outcome the effect measure(s) (e.g., risk ratio, mean difference) used in the synthesis or presentation of results.	3
Synthesis methods	13a	Describe the processes used to decide which studies were eligible for each synthesis (e.g. tabulating the study intervention characteristics and comparing against the planned groups for each synthesis (item #5)).	3
	13b	Describe any methods required to prepare the data for presentation or synthesis, such as handling of missing summary statistics or data conversions.	3
	13c	Describe any methods used to tabulate or visually display results of individual studies and syntheses.	3
	13d	Describe any methods used to synthesize results and provide a rationale for the choice(s). If meta-analysis was performed, describe the model(s), method(s) to identify the presence and extent of statistical heterogeneity, and software package(s) used.	NA
	13e	Describe any methods used to explore possible causes of heterogeneity among study results (e.g., subgroup analysis, meta-regression).	NA
	13f	Describe any sensitivity analyses conducted to assess robustness of the synthesized results.	NA
Reporting bias assessment	14	Describe any methods used to assess risk of bias due to missing results in a synthesis (arising from reporting biases).	3
Certainty assessment	15	Describe any methods used to assess certainty (or confidence) in the body of evidence for an outcome.	3
**RESULTS**			
Study selection	16a	Describe the results of the search and selection process, from the number of records identified in the search to the number of studies included in the review, ideally using a flow diagram.	3
	16b	Cite studies that might appear to meet the inclusion criteria, but which were excluded, and explain why they were excluded.	3 (cfr. flow chart)
Study characteristics	17	Cite each included study and present its characteristics.	3–4
Risk of bias in studies	18	Present assessments of risk of bias for each included study.	4, 11 (cfr. Risk of bias table)
Results of individual studies	19	For all outcomes, present, for each study: (a) summary statistics for each group (where appropriate) and (b) an effect estimate and its precision (e.g. confidence/credible interval), ideally using structured tables or plots.	7–10 (cfr. Table of evidence)
Results of syntheses	20a	For each synthesis, briefly summarise the characteristics and risk of bias among contributing studies.	4–6
	20b	Present results of all statistical syntheses conducted. If meta-analysis was done, present for each the summary estimate and its precision (e.g., confidence/credible interval) and measures of statistical heterogeneity. If comparing groups, describe the direction of the effect.	4–6
	20c	Present results of all investigations of possible causes of heterogeneity among study results.	3–4 (cfr. Study characteri stics)
	20d	Present results of all sensitivity analyses conducted to assess the robustness of the synthesized results.	NA
Reporting biases	21	Present assessments of risk of bias due to missing results (arising from reporting biases) for each synthesis assessed.	4, 11 (cfr. Risk of bias table)
Certainty of evidence	22	Present assessments of certainty (or confidence) in the body of evidence for each outcome assessed.	4, 11 (cfr. Risk of bias table)
**DISCUSSION**			
Discussion	23a	Provide a general interpretation of the results in the context of other evidence.	13–14
	23b	Discuss any limitations of the evidence included in the review.	14–15
	23c	Discuss any limitations of the review processes used.	14–15
	23d	Discuss implications of the results for practice, policy, and future research.	15
**OTHER INFORMATION**			
Registration and protocol	24a	Provide registration information for the review, including register name and registration number, or state that the review was not registered.	2
	24b	Indicate where the review protocol can be accessed, or state that a protocol was not prepared.	2
	24c	Describe and explain any amendments to information provided at registration or in the protocol.	2
Support	25	Describe sources of financial or non-financial support for the review, and the role of the funders or sponsors in the review.	NA
Competing interests	26	Declare any competing interests of review authors.	NA
Availability of data, code and other materials	27	Report which of the following are publicly available and where they can be found: template data collection forms; data extracted from included studies; data used for all analyses; analytic code; any other materials used in the review.	NA

From: Page MJ, McKenzie JE, Bossuyt PM, Boutron I, Hoffmann TC, Mulrow CD, et al. The PRISMA 2020 statement: an updated guideline for reporting systematic reviews. BMJ 2021;372:n71. doi: 10.1136/bmj.n71 For more information, visit: http://www.prisma-statement.org/

## Appendix A2. Literature Search Strategy

### PubMed

(fatigue OR fatigability OR exertional OR Exhaustion OR exhausted OR depletion) AND (Biomechanics OR Kinematics OR angles OR kinetics OR “Electromyography”[Mesh] OR moment OR power OR mechanics) AND (“Plyometric exercise"[Mesh] OR Landing OR Jumping OR jump OR hop OR ”Single-legged function test” OR cutting)

### Embase/CINAHL/SPORTDiscus

(fatigue OR fatigability OR exertional OR exhaustion OR exhausted OR depletion) AND (biomechanics OR kinematics OR angles OR kinetics OR electromyography OR moment OR power OR mechanics) AND ('plyometric exercise' OR landing OR jumping OR jump OR hop OR 'single-legged function test' OR cutting)

### Web of Science

TS=((fatigue OR fatigability OR exertional OR exhaustion OR exhausted OR depletion) AND (biomechanics OR kinematics OR angles OR kinetics OR electromyography OR moment OR power OR mechanics) AND ('plyometric exercise' OR landing OR jumping OR jump OR hop OR 'single-legged function test' OR cutting))

## Appendix A3. Modified Downs and Black Checklist

### Reporting

1. Is the hypothesis and aim(/objective) of the study clearly described?

YES: 1

NO: 0

2. Are the main outcomes to be measured clearly described in the Introduction and/or Methods?

YES: 1: Clearly description of kinematics (plane and joint) and/or EMG, their units and phase of landing

NO: 0: No clearly description

3. Are the characteristics of the participants included in the study clearly described?

YES: 1: Clearly description of selection criteria

NO: 0: None of the above criteria is present or not mentioned

4. Are the interventions of interest clearly described?

YES: 1: Clear description of the fatigue protocol (sets/repetitions and clear description of criteria to determine point of fatigue)

NO: 0: No clear description of the fatigue protocol

5. Are the distributions of principal confounders in each group of subjects to be compared clearly described?

YES: 2: Clear description of

• Physical activity (intensity or frequency)

• Level of fatigue (subjective or objective)

• Recovery during post-fatigue testing

PARTIALLY: 1: Clear description of at least one principal confounder

NO: 0

6. Are the main findings of the study clearly described?

YES: 1: Quantitative description of the main outcome data

NO: 0: No quantitative description of the main outcome data

7. Does the study provide estimates of the random variability in the data for the main outcomes?

YES: 1: In normally distributed data: ‘mean ± SD or CI’; non-normally distributed data: inter-quartile range; or if distribution of data is not described

NO: 0: When the use of ‘mean ± SD’ in non-normally distributed data

8: Have all important adverse events that may be a consequence of the intervention been reported?

YES: 1: Measurement of the adverse event due to the fatigue protocol (VAS, complaints,…) or a list of possible adverse events

NO: 0: No measurement of the adverse event due to the fatigue protocol

10. Have actual probability values been reported (e.g., 0.035 rather than < 0.05) for the main outcomes except where the probability value was less than 0.001?

YES: 1

NO: 0

### External validity

11. Were the selected subjects representative of the entire population from which they are recruited?

YES: 1: Participants are representative for the target population described in the aims of the study

NO: 0

Unable to determine: 0

12. Were those subjects who were prepared to participate representative of the entire population from which they were recruited?

YES: 1: The proportion of those asked who agreed should be stated

NO: 0: No statement

Unable to determine: 0

### Internal validity – bias

16. If any of the results of the study were based on “data dredging”, was this made clear?

YES: 1

NO: 0

Unable to determine: 0

18. Were the statistical tests used to assess the main outcomes appropriate?

YES: 1

NO: 0

Unable to determine: 0

20. Were the main outcome measures used accurately (valid and reliable)?

• Regarding kinematics as the main outcome measure

YES: 1: 3D kinematics

NO: 0: 2D kinematics

Unable to determine: 0

• Regarding muscular activity (measured by EMG) as the main outcome measure

YES: 1: Detailed description of data processing

NO: 0: No detailed description of data processing

Unable to determine: 0

• In case of kinematics and muscular activity (measured by EMG) as the main outcome measure

YES: 1: 3D kinematics and detailed description of EMG data processing

NO: 0: 2D kinematics and/or no detailed description of data processing

Unable to determine: 0

### Internal validity – confounding (selection bias)

25. Was there adequate adjustment for confounding in the analyses from which the main findings were drawn?

YES: 1: A statistical correction for the confounding factors was made

NO: 0: Only a list of confounders was reported

Unable to determine: 0
